# Diabetes mellitus impacts risk of macrovascular invasion in patients undergoing transplantation for hepatocellular carcinoma

**DOI:** 10.1186/1471-230X-13-9

**Published:** 2013-01-14

**Authors:** Gregory C Connolly, Saman Safadjou, Randeep Kashyap, Rui Chen, Mark S Orloff, Aram F Hezel

**Affiliations:** 1 The Department of Medicine, James P. Wilmot Cancer Center, and, University of Rochester, Rochester, NY, USA; 2 The Division of Solid Organ Transplantation and Hepatobiliary Surgery, University of Rochester, Rochester, NY, USA; 3 The Department of Biostatistics and Computational Biology, University of Rochester, Rochester, NY, USA

**Keywords:** Diabetes mellitus, Hepatocellular carcinoma, Vascular invasion

## Abstract

**Background:**

Diabetes mellitus (DM) is identified as a negative prognostic indicator in hepatocellular carcinoma (HCC), though the basis for this is unknown.

**Methods:**

This is a retrospective analysis of a prospectively collected database of 191 HCC patients treated at the University of Rochester Medical Center (URMC) with orthotopic liver transplantation between 1998–2008. Clinical characteristics were compared between patients with and without DM prior to liver transplantation and logistic regression analyses were conducted to assess the effect of DM on clinical outcomes including vascular invasion.

**Results:**

Eighty-four of 191 (44%) transplanted patients had DM at time of transplantation. An association of DM with invasive disease was found among transplanted HCC patients where histologically confirmed macrovascular invasion was found in 20.2% (17/84) of diabetics compared to 9.3% of non-diabetics (10/107) (p=0.032). This difference also remained significant when adjusting for tumor size, number of nodules, age, obesity and etiologic risk factors in multivariate logistic regression analysis (OR=3.2, p=0.025).

**Conclusions:**

DM is associated with macrovascular invasion among a cohort of transplanted HCC patients.

## Background

Hepatocellular carcinoma (HCC) is the fifth most common cancer and the third most common cause of cancer related death worldwide [1]. The incidence in the United States has tripled over the past three decades [2] and is estimated to increase further over the next two decades largely due to the increasing prevalence of hepatitis C infection [3-5]. Established risk factors for HCC include chronic viral hepatitis and alcohol abuse include tobacco smoking [6], non-alcoholic steatohepatitis (NASH) [7], autoimmune hepatitis [8], obesity [9], aflatoxin exposure [10], and diabetes mellitus (DM) [11-13].

There is evidence to support a role for DM in the development and progression of HCC. An association between DM and HCC has been demonstrated by population based cohort studies of diabetic patients [11-13] and case–control studies of HCC patients [14-16]. DM negatively impacts survival in early stage HCC patients undergoing resection and other local treatments [17-20] but there is limited data on the impact of DM in the setting of transplanted HCC patients and little is known about the mechanism by which diabetes causes decreased survival. The current study examines a prospectively collected cohort of transplanted HCC patients. As all patients had complete tumor pathologic analysis we focused on pathologic features that might account poorer prognosis in HCC with DM.

## Methods

This study was approved by the University of Rochester Institutional Review Board (IRB) and is a retrospective analysis of a prospectively collected database. HCC patients who received cadaveric liver transplantation at the University of Rochester medical center (URMC) between January 1998 and December 2008 were identified through the prospectively collected solid organ transplant database. (n=191) Criteria for transplantation included absence of overt tumor thrombus and tumor metastasis, adequate physical health and general functional status, and adequate social support. All patients listed for liver transplantation at URMC are prospectively followed as part of the United Network of Organ Sharing (UNOS) program eligibility requirements. Patients are entered into this database at the time transplantation occurs. Baseline demographic data, date of HCC diagnosis, past medical history, laboratory data, imaging information and explant pathologic data are entered at the time of transplantation. Patients were considered to have alcohol related cirrhosis as an etiologic risk factor if there was a history of at least 3 alcoholic beverages per day for a minimum of 6 months. Post-transplant laboratory data, imaging data and information pertaining to recurrence, survival, and cause of death was collected and entered prospectively.

Pre-transplant DM status was determined by thorough retrospective chart review. Post-transplant DM status was not obtained or included in this analysis. Patients were considered to have DM if the most recent history and physical or office visit note prior to liver transplantation indicated regular use of oral hypoglycemic agents or insulin as an outpatient or a history of DM in the past medical history section.

Pathologic reporting on transplanted HCC is standardized and includes tumor size, grade, and comprehensive information on macrovascular and microvascular invasion [21]. The number of tumors and tumor sizes were obtained from pathology reports of surgical specimens. Patients were considered to have NASH as a potential etiologic risk factor only if there was clear evidence of steatohepatitis on pathologic review of the explant specimen. Macrovascular and microvascular invasion were defined pathologically as previously described [22]. Macrovascular invasion was defined as vascular invasion of either major portal veins or hepatic veins, and microvascular invasion as microscopic vascular invasion of the tumor by small vessels within the parenchyma of the liver. Body mass index (BMI) was prospectively collected in all patients listed for transplant and the value was calculated based on height and weight at closest available time-point prior to transplantation.

Patient demographics and clinical characteristics were summarized. Continuous variables were compared between non-diabetic and diabetic groups using t-tests. Chi-square tests were used to compare the difference between the two groups for categorical variables in each cohort. Similarly, the associations between macrovascular invasion and DM as well as post-transplant HCC recurrence and DM were evaluated in the transplant cohort using multivariate logistic regressions. We included total tumor diameter and multinodular disease in the multivariate models a-priori because they are known risk factors for macrovascular invasion and HCC recurrence [23]. Variables including age, BMI and etiologic risk factors (NASH, hepatitis C and hepatitis B) were included in the multivariate models because univariate testing revealed significant differences (p<0.05) in distribution between the diabetic and non-diabetic cohorts. Mortality was estimated by the method of Kaplan-Meier and differences between groups were compared using log-rank test in each cohort. The transplant program at the University of Rochester has a quality assessment committee. Cause of death was determined by transplant committee multidisciplinary consensus in a prospective fashion when cause of death was ascertainable. Hazard ratios for the relevant clinical factors were assessed using multivariate Cox regressions. Tests were two-sided with p<0.05 considered statistically significant. All statistical analyses were carried out using SPSS.

## Results

### Patient characteristics of transplanted cohort

The mean age of the transplanted population was 57.2 ± 8.6 and 85.9% were males (Table 1). The most common underlying etiologic risk factors for cirrhosis in the transplanted cohort were chronic hepatitis C (54.5%), alcohol abuse (24.6%), NASH (7.3%) and chronic hepatitis B (6.2%). Less common risk factors included hemochromatosis (9 patients), alpha-1 antitrypsin deficiency (2 patients), autoimmune hepatitis (2 patients), and biliary atresia (1 patient). Five patients (2.6%) had more than one known risk factor for cirrhosis.

**Table 1:** Patient characteristics

**Category**	**Total (n=191)**	**Diabetic (n=84)**	**Non-Diabetic (n=107)**	**P Value**
Age (Mean ± StDev)	57.2 ± 8.6	60.7 ± 7.2	55.03 ± 8.9	< 0.0001
>65 years old (%)	40 (20.9%)	24 (28.6%)	16 (15.0%)	0.022
Sex (% Male)	164 (85.9%)	75 (89.3 %)	89 (83.2 %)	0.23
Body mass index > 30	78 (40.8%)	40 (47.6%)	42 (39.3%)	0.017
Etiologic Risk Factors *				
Alcohol abuse	47 (24.6%)	18 (21.4%)	29 (27.1%)	0.37
Hepatitis C	104 (54.5%)	37 (44%)	67 (62.6%)	0.011
Hepatitis B	12 (6.2%)	5 (5.9%)	7 (6.5%)	0.86
Non-alcoholic steatohepatitis	14 (7.3%)	11 (13.1%)	3 (2.8%)	0.007
Cryptogenic	20 (10.5%)	13 (15.5%)	7 (6.5%)	0.057
Tumor Characteristics				
Total tumor diameter (mean)+	5.42	5.81	5.125	0.29
Multinodular	107 (56%)	46 (54.8%)	61 (57%)	0.77
Macrovascular Invasion	27 (14.1%)	17 (20.2%)	10 (9.3%)	0.032
Microvascular Invasion Only	33(17.4%)	15 (17.9%)	18 (17.0%)	0.87
Portal Vein Thrombosis	33 (17.3%)	19 (22.6%)	14 (13.1%)	0.12
Laboratory Parameters++				
MELD score (Mean ± SD)	15.91 ± 7.8	14.69 ± 7.19	16.86 ± 8.27	0.062
INR (Mean ± SD)	1.51 ± 0.52	1.37 ± 0.35	1.64 ± 0.59	0.001
Albumin (Mean ± SD)	3.3 ± 0.73	3.34 ± 0.82	3.27 ± 0.65	0.55
Bilirubin (Mean ± SD)	4.12 ± 5.69	3.34 ± 4.96	4.73 ± 6.16	0.071
Creatinine (Mean ± SD)	1.27 ± 0.81	1.36 ± 0.94	1.21 ± 0.70	0.20
Alpha fetoprotein IU/ml (median)	16.5+++	16.5	16.5	0.63
Alpha fetoprotein IU/ml (mean)		978	542	0.35
HCC treatment				
Chemoembolization	5(2.6%)	3(3.6%)	2 (1.9%)	0.76
Radiation	11(5.8%)	4 (4.8%)	7 (6.5%)	0.76
Ablation	7 (3.7%)	3 (3.6%)	4 (3.7%)	0.95
Transplant wait list time (mean number of months)		5.9 months	7.5 months	0.43

* Five patients had more than one etiologic risk factor.

+ seven missing values.

++twenty one patients from pre-MELD era were missing laboratory values and MELD score.

+++ two missing values.

Forty four percent (84/191) of the transplanted patients had DM at the time of liver transplantation, and of those with DM 11.9% (10/84) were diet controlled, 28.6% (21/84) used oral sulfonylurea medication, 11.9% (10/84) used non-sulfonylurea medication, and 51.2% (43/84) used insulin. There was a non-significant trend toward increased prevalence of DM in more recent years. Patients with DM were significantly older at the time of liver transplantation (60.7 vs 55.0, p<0.0001), and the prevalence of NASH was significantly higher in DM patients (13.1% vs 2.8%, p=0.007) (Table 2). Non-DM patients had a significantly higher INR at the time of transplant (1.64 vs 1.37, p=0.001), and a higher proportion of non-DM patients had chronic hepatitis C (62.6% vs 44.0%, p=0.011). All other characteristics and pre-transplant mean laboratory values were similar between patients with and without DM. Similar proportions of each group received pre-transplant treatment such as chemoembolization, ablation, radiation and resection. In the transplanted cohort, the average total tumor diameter was similar between non-DM and DM patients (5.12 cm vs 5.81 cm, p=0.294), and similar proportions had a largest tumor that was >5cm in diameter (16.8% of non-DM vs 23.8% DM, p=0.274). Fifty seven percent of non-DM patients had multinodular disease compared to 54.8% of patients with DM in the transplant population (p=0.771). Portal vein thrombosis was found on histologic review of the liver explant in 13.1% of non-diabetics and 22.6% of diabetics (p=0.122).

**Table 2:** Multivariate analysis for macrovascular invasion among transplanted patients

**Characteristics**	**Odds Ratio**	**95% Confident Limits**		**P value**
DM	3.24	1.16	9.06	0.025
Total Tumor Diameter	1.24	1.11	1.39	0.0001
Multinodular vs Uninodular	2.05	0.63	6.72	0.23
Age > 65	1.26	0.35	4.63	0.72

OR’s were adjusted for BMI (≥30 vs <30) and etiologic risk factors (HCV, HBV, NASH, and alcoholic cirrhosis).

### Impact of DM on HCC macrovascular invasion

The presence of DM was associated with a significantly higher rate of histologically confirmed macrovascular in the cohort of HCC patients receiving liver transplantation. Seventeen of 84 (20.2%) patients with DM had histologic evidence of gross vascular invasion compared to 10 of 107 (9.4%) non-DM patients (p=0.034). Only one of these subjects had a suggestion of possible macrovascular invasion on pre-transplant imaging. All but one patient with evidence of macrovascular invasion also had histologic evidence of microvascular invasion. Thirty three patients (17.2%) had evidence of microvascular invasion without macrovascular invasion, and the rate was equal in patients with and without DM (17.9% vs 17.0%, p=0.82). In multivariate logistic regression analysis adjusting for total tumor diameter, number of nodules, age, BMI>30 and etiologic risk factors the presence of DM predicted for an increased risk of macrovascular invasion (OR=3.2, p=0.025) (Table 2). If NASH and BMI are taken out of the model DM remains a significant independent predictor of macrovascular invasion (OR=3.19, p0.034). In addition we examined the model with the additional interaction term of diabetes and macrovascular invasion in the multivariate regression analysis and the effect was not significant (p=0.6407). Therefore, we did not include the term in our final model.

### DM and post-transplant HCC recurrence

Post-transplant HCC recurrence among patients with a history of DM was not significantly different than recurrence among patients without DM (28.6% (24/84) vs 19.6% (21/107), ( p=0.15). In multivariate logistic regression analysis including DM, vascular invasion, total tumor diameter, number of nodules, age, and etiologic risk factors both microvascular invasion (OR3.7, p=0.009) and macrovascular invasion (OR=32.5, p=<0.0001) but not DM (OR=0.96, p=0.9) were associated with an increased risk of post-transplant HCC recurrence (Table 3). The highest rates of post-transplant recurrence were seen in diabetic patients receiving insulin. Thirty percent (13/43) of patients with DM receiving insulin recurred after transplantation compared to 26.8% (11/41) of patients with DM not receiving insulin and 19.6% (21/107) of non-diabetics.

**Table 3:** Multivariate analysis of post-transplant HCC recurrence

**Characteristics**	**Odds Ratio**	**95% Confidence Limits**		**P value**
DM	0.96	0.39	2.34	0.93
Vascular Invasion				
Microvascular only vs. None	3.71	1.39	9.91	0.009
Macrovascular vs. None	32.50	8.91	118.55	< 0.0001
Total Tumor Diameter	1.07	0.96	1.18	0.21
Age >65	0.55	0.17	1.79	0.32

OR’s were adjusted for BMI (≥30 vs <30) and etiologic risk factors (HCV, HBV, NASH, and alcoholic cirrhosis).

### Impact of diabetes on survival in HCC

At the time of this analysis 50.3% (96/191) of the transplanted patients had died over a median follow up time of 63.4 months. The median survival as estimated by Kaplan meier survival analysis of the transplanted non-DM patients was 78.3 months compared to 31.1 months in transplanted patients with DM (p=0.2). The cause of death was known for 85 patients with most common causes of death being recurrent HCC (41.2%, 35/85), infection (34.1%, 29/85) and cardiovascular disease (14.1%, 12/85). In the multivariate Cox regression analysis including age, total tumor diameter, multinodular vs uninodular disease and DM, macrovascular invasion predicted for worse survival in the transplant cohort (HR=4.7, p=<0.0001) (Table 4).

**Table 4:** Multivariate analysis of post-transplant overall survival

**Characteristics**	**Hazard Ratio**	**95% Confident Limits**		**P value**
Macrovascular Invasion	4.73	2.72	8.23	<0.0001
DM	1.06	0.69	1.62	0.76
Total Tumor Diameter	0.98	0.94	1.03	0.47
Multinodular	1.23	0.78	1.94	0.37
Age >65	1.18	0.72	1.93	0.51

HR’s were adjusted for BMI (≥30 vs <30) and etiologic risk factors (HCV, HBV, NASH, and alcoholic cirrhosis).

## Discussion

This analysis suggests that DM is associated with an increased incidence of histologically confirmed vascular invasion in a cohort of HCC patients receiving liver transplantation. DM remained a significant predictor of macrovascular invasion when adjusting for tumor size, number of nodules, age, obesity and etiologic risk factors in multivariate analysis. Among pathologic features of HCC macrovascular invasion is identified as the strongest predictor of post-transplant recurrence of HCC and survival, a finding consistent with previous reports [24,25].

A number of studies have evaluated the impact of DM on HCC incidence, prognosis, and disease behavior. Patients with DM have up to a 4-fold increased risk of developing HCC compared to patients without DM [11-16]. Studies of early stage HCC patients undergoing liver resection demonstrated significantly decreased overall survival and recurrence free survival in diabetic patients compared to non-diabetics by multivariate analysis [18,19]. Other studies in early stage HCC patients with DM treated with resection have demonstrated decreased survival in some subgroups [17,20].

The impact of DM on pathologic parameters has been evaluated in colorectal cancer where it is suggested to influence pathologic stage [26] and in breast cancer where DM is associated with estrogen receptor negativity. [27,28] Macrovascular invasion [24] and microvascular invasion [25] have been identified as key negative prognostic indicators in HCC, findings which were echoed in the current study. Among patient and tumor characteristics that could be indentified prior to transplantation tumor size and the presence of DM were significantly associated with macrovascular invasion. Macrovascular invasion was the strongest predictor of disease recurrence following transplantation and significantly associated with risk of post-transplant death.

Several potential explanations can be offered regarding the association between DM and HCC development and progression. Stage migration, delayed diagnosis, or less stringent screening may be factors although our analysis showing equal waiting list times would refute this argument. DM may have a direct causative role in HCC development and progression possibly through promotion of carcinogenesis, tumor growth, and invasiveness. These direct effects may be mediated by alterations in gene transcription induced by increased levels of glucose [29], insulin, or counter-regulatory hormones such as growth hormone and insulin-like growth factor. An etiologic role for increased insulin levels is supported by epidemiologic studies showing a higher rate of HCC development in diabetic cirrhotics treated with insulin compared to those treated with oral hypoglycemic agents [30]. Also, diabetic patients may have alterations in hepatocyte expression of surface receptors such as insulin receptor or insulin-like growth factor receptor that increase susceptibility to unidentified tumor promoting factors. Recent studies have demonstrated increased expression and activity of ILGFR-1 in HCC tumors [31], and inhibition of these receptors in animal models results in decreased metastatic potential of HCC tumor cell lines [32].

There are several limitations to this study. First the presence of DM was determined based on medical history or medication use rather than by fasting glucose or hemoglobin A1C measurement. Thus, the presence of physiologic glucose intolerance is likely underestimated. The lack of reliable data on the timing of DM onset relative to diagnosis of cirrhosis and HCC hinder our ability to confer a causal relationship. We were not able to examine the potential impact of DM on histologic grade or histologic grade as a potential confounder on the relationship between DM and vascular invasion because histologic grade was not adequately captured in this database. Also, this study was exploratory and hypothesis generating in nature and confined to a single institution. It is possible that the observed associations are due to chance. Confirmatory studies in larger cohorts including multiple institutions are needed. Given that this is a tertiary liver transplant referral center there is potential for referral bias. Lastly, we did not observe a significant difference in overall survival although we do note a trend toward decreased survival as indicated by the Kaplan meier estimates. Patients with transplanted HCC die from causes other than recurrent or uncontrolled HCC such as liver failure, infection, bleeding, and post-operative complications in the setting of transplantation and there is considerable variation in survival times as indicated by the large standard deviations in mean survival times. It seems likely that any direct effect of DM on HCC-related mortality would require a larger cohort.

## Conclusions

Despite the limitations this study offers evidence that DM is associated with increased vascular invasion in HCC. These novel findings, if confirmed, could have clinical implications, and suggest clinicians may need to consider the impact of DM when selecting patients for liver transplantation. The incidence of glucose intolerance and DM in patients with liver cirrhosis is 60-80% and 20-60% respectively [33] so it is imperative that we understand how to best manage DM in the setting of cirrhosis and HCC. The current study suggests further research is required to better understand the interaction between DM and disease pathogenesis and outcomes in HCC.

## Competing interest

The authors declare that they have no competing interests.

## Authors’ contributions

GC and SS carried out the retrospective data collection. GC drafted the manuscript. All authors read and approved the final manuscript.

## Pre-publication history

The pre-publication history for this paper can be accessed here:

http://www.biomedcentral.com/1471-230X/13/9/prepub
